# Sequential Anabolic and Antiresorptive Treatment Promotes Medication‐Related Osteonecrosis of the Jaw in Mice

**DOI:** 10.1111/odi.70417

**Published:** 2026-07-19

**Authors:** Seo‐Yeong An, Hae‐Seo Park, Reuben H. Kim, Moon‐Young Kim

**Affiliations:** ^1^ Department of Oral and Maxillofacial Surgery College of Dentistry, Dankook University Cheonan Republic of Korea; ^2^ The Shapiro Family Laboratory of Viral Oncology and Aging Research Los Angeles California USA; ^3^ UCLA Jonsson Comprehensive Cancer Centre Los Angeles California USA

**Keywords:** denosumab, healing, MRONJ, osteoclast, romosozumab

## Abstract

**Objective:**

To investigate the effects of sequential administration of romosozumab and denosumab on post‐extraction socket healing and osteonecrosis development in a murine tooth extraction model.

**Methods:**

Forty female mice were randomly assigned to four groups: control (Ctrl), romosozumab (Rm), denosumab (Dmab), and sequential romosozumab followed by denosumab (Rm + Dmab). After drug administration, bilateral maxillary first molars were extracted. Extraction socket healing was evaluated by micro‐computed tomography, histological analysis, and immunohistochemistry for receptor activator of nuclear factor κB ligand (RANKL), osteoprotegerin (OPG), and sclerostin.

**Results:**

The Rm + Dmab group exhibited significantly less new bone formation and more necrotic bone in the extraction sockets than the other groups, accompanied by a marked reduction in osteoclast numbers. The Dmab‐only group showed a lower RANKL/OPG ratio. No significant differences in sclerostin expression were observed among groups.

**Conclusions:**

Sequential administration of romosozumab followed by denosumab severely impaired extraction socket healing and induced osteonecrotic lesions in mice, resembling medication‐related osteonecrosis of the jaw (MRONJ). These findings suggest that such sequential antiresorptive therapy may increase the risk of MRONJ in patients undergoing dental extractions.

## 1 Introduction

Medication‐related osteonecrosis of the jaw (MRONJ) is a severe adverse effect associated with the use of antiresorptive and antiangiogenic agents, characterized by persistent, non‐healing necrotic bone exposure in the maxillofacial region (Ruggiero et al. 2022). The risk of MRONJ is influenced by the underlying disease, the specific agent used, treatment duration, and local oral factors. In particular, MRONJ occurs more frequently in cancer patients receiving high‐dose antiresorptive therapy than in patients treated for osteoporosis, with reported risk estimates generally below 5% in malignancy populations and below 0.05% in osteoporosis populations (Ruggiero et al. 2022).

Bisphosphonates such as zoledronate have long been implicated in the development of MRONJ. In recent years, newer therapeutic agents, including denosumab, a monoclonal antibody against receptor activator of nuclear factor κB ligand (RANKL), and romosozumab, a sclerostin‐neutralizing antibody, have also been associated with this complication (Ruggiero et al. 2022; Taylor et al. 2010). Denosumab is a widely used antiresorptive that inhibits osteoclast‐mediated bone resorption by targeting the RANK/RANKL/osteoprotegerin (OPG) pathway (Kostenuik et al. 2009). By contrast, romosozumab is an anabolic bone‐forming agent that neutralizes sclerostin and thereby activates Wnt/β‐catenin signaling, while also secondarily reducing bone resorption (Taylor et al. 2016). The introduction of these agents has expanded the spectrum of medications linked to MRONJ and raised new concerns about their effects on craniofacial bone health.

Although romosozumab is primarily recognized for its anabolic effects on bone, recent clinical reports and regulatory reviews have suggested that it may present a potential, albeit lower, risk of MRONJ compared to traditional bisphosphonates or denosumab (Kim et al. 2022; Ruggiero et al. 2022). However, the pathophysiological mechanisms by which romosozumab, either alone or administered sequentially with denosumab, might influence MRONJ development and impair bone healing remain unclear. This uncertainty is clinically significant, as patients are increasingly exposed to sequential osteoporosis therapies that include both anabolic and antiresorptive phases.

Indeed, the 2020 clinical practice guidelines from the American Association of Clinical Endocrinology (AACE) recommend a sequential approach to osteoporosis treatment: initiating therapy with an anabolic agent followed by an antiresorptive drug for postmenopausal women at very high risk of osteoporotic fracture (Camacho et al. 2020). Such strategies underscore the importance of understanding how the sequential administration of anabolic and antiresorptive medications affects bone wound healing and influences the risk of developing MRONJ, particularly in the context of dental extractions or oral surgery.

Despite these considerations, experimental studies investigating the effects of sequential anabolic and antiresorptive therapy on extraction socket healing and MRONJ pathogenesis remain scarce. Recently, our group demonstrated that sequential administration of romosozumab followed by zoledronate exacerbated MRONJ development in a murine tooth extraction model, with the combined regimen resulting in more severe bone necrosis and greater suppression of osteoclast activity than zoledronate alone (Park et al. 2026). However, the effects of romosozumab followed by denosumab on extraction socket healing and MRONJ development have not been investigated, despite this combination being increasingly used in clinical practice. To address this knowledge gap, the present study investigated alveolar bone healing after maxillary molar extractions in a murine model under different treatment regimens, including sequential administration of romosozumab and denosumab. As an initial exploratory investigation, we examined healing outcomes through comparative radiographic, histological, and histomorphometric analyses aiming to clarify the impact of these therapeutic regimens on post‐extraction healing and MRONJ‐related changes (Figure 1).

**FIGURE 1:**
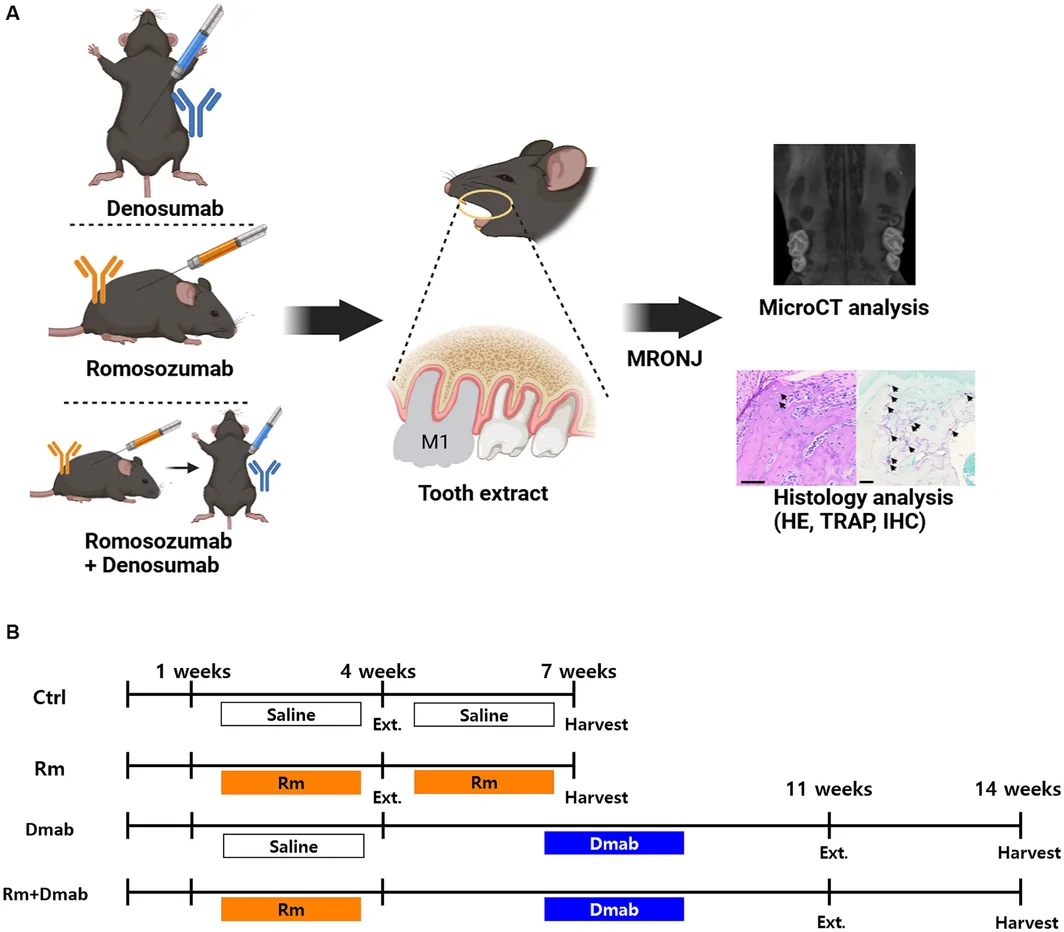
Schematic overview of the experimental design, methodology, and timetable of the experimental protocol. (A) Forty 8‐week‐old female C57BL/6J mice were enrolled and randomly assigned to receive one of four treatments: Intraperitoneal injections of a murine anti‐RANKL antibody (denosumab equivalent; clone CD254, Bio X Cell, USA) at 250 μg/kg twice weekly; subcutaneous injections of romosozumab at 10 mg/kg once weekly; a sequential combination of romosozumab followed by the anti‐RANKL antibody; or saline as control. Bilateral maxillary first molars were extracted in all mice. Three weeks after extraction, the animals were sacrificed and tissue samples were harvested. Subsequent analyses included radiographic assessment, histological staining (H&E: Hematoxylin and Eosin; TRAP: Tartrate‐resistant acid phosphatase), and immunohistochemical evaluation of RANKL (Receptor activator of nuclear factor kappa‐B ligand), OPG (Osteoprotegerin), and sclerostin (SOST) protein expression. (B) Mice were allocated to one of four groups: Ctrl (saline for 3 weeks), Rm (romosozumab for 3 weeks), Dmab (saline for 3 weeks followed by anti‐RANKL antibody for 7 weeks), and Rm + Dmab (romosozumab for 3 weeks followed by anti‐RANKL antibody for 7 weeks). Maxillary first molars were extracted at Week 4 in the Ctrl and Rm groups, and at Week 11 in the Dmab and Rm + Dmab groups. All groups received an additional 3 weeks of their respective treatments following tooth extraction. Orange bars represent periods of romosozumab administration, and blue bars indicate anti‐RANKL antibody administration.

## 2 Materials and Methods

### 2.1 Animal Care and Experimental Design

Forty female C57BL/6 mice, aged 8 weeks, were used in this study, following the guidelines of the Dankook University Institutional Animal Care and Use Committee (DKU‐23‐004). After 1 week of acclimation, mice were randomly assigned to four groups: Control (Ctrl), Romosozumab + Denosumab (Rm + Dmab), Denosumab only (Dmab), Romosozumab only (Rm) (Figure 1).

Mice were housed in plastic cages with corn bedding (four per cage) under controlled conditions (22°C, 50% relative humidity, 12‐h light/dark cycle) and had ad libitum access to food and water throughout the experiment.

### 2.2 Drug Administration

The Control group received saline subcutaneously once weekly for 3 weeks before tooth extraction. The Rm group received romosozumab (Evenity, Amgen, Thousand Oaks, CA, USA) subcutaneously at 10 mg/kg once weekly for 3 weeks before extraction. The Dmab group received saline for 3 weeks, then a murine anti‐RANKL antibody (denosumab equivalent; clone CD254, Bio X Cell, Lebanon, USA) intraperitoneally at 250 μg/kg twice a week for 7 weeks before extraction. The Rm + Dmab group received romosozumab at the same dose and schedule as above for 3 weeks, followed by the anti‐RANKL antibody at the same dose and schedule as above for 7 weeks before extraction. After tooth extraction, all groups continued their respective drug regimens for an additional 3 weeks before sacrifice (Figure 1).

### 2.3 Tooth Extraction Procedure

Mice were anesthetized with an intraperitoneal injection of tiletamine‐zolazepam (Zoletil 50, Virbac, Carros, France) at 30 mg/kg and xylazine (Rompun, Bayer Korea, Seoul, Korea) at 10 mg/kg. Both maxillary first molars were extracted. Meloxicam (Metacam 2%, Boehringer Ingelheim, Seoul, Korea) was administered subcutaneously at 1 mg/kg for postoperative pain relief. The maxillae were harvested 3 weeks following extraction, photographed under consistent magnification and lighting conditions, and fixed in 10% buffered formalin.

### 2.4 Micro‐Computed Tomography (μCT) Analysis

Harvested maxillae were scanned using a Skyscan 1176 micro‐CT system (Bruker‐Microct, Kontich, Belgium) at 50 kV and 500 μA, with a pixel size of 8.96 μm. Images were reconstructed using NRecon software and analyzed with CTAn (Bruker‐micro‐CT). Binarization thresholds were empirically determined based on the grayscale histograms of representative samples, and a threshold range of 0–80 was applied uniformly across all groups to ensure consistent segmentation. Bone volume (BV), tissue volume (TV), and bone volume fraction (BV/TV) within the extraction socket were calculated.

### 2.5 Histological and Histomorphometric Analysis

#### 2.5.1 Hematoxylin and Eosin (H&E) Staining

Fixed maxillae were decalcified in 5% EDTA (pH 7.4) at 4°C for 6 weeks, dehydrated, embedded in paraffin, sectioned at 5 μm, and stained. The region of interest (ROI) included the extraction site and adjacent bone. The number of empty lacunae and necrotic bone area (defined as five or more adjacent empty lacunae) were quantified per mm^2^ and as a percentage of ROI area. All histomorphometric analyses were performed using ImageJ software (NIH, Bethesda, MD, USA; version 1.54 g) by a blinded examiner.

#### 2.5.2 Tartrate‐Resistant Acid Phosphatase (TRAP) Staining

Paraffin sections were treated with 50% acetone/ethanol, then incubated with TRAP staining solution (Takara, Japan) at 37°C for 10 min, followed by toluidine blue counterstain. TRAP‐positive multinucleated cells (≥ 3 nuclei) were counted per mm^2^ in the ROI using ImageJ software by a blinded examiner.

#### 2.5.3 Immunohistochemistry (IHC)

Immunohistochemical analysis was used to assess the spatial distribution and relative expression of target proteins within the bone tissue. For immunohistochemistry, sections were deparaffinized, and antigen retrieval was performed in citrate buffer (pH 6.0). Endogenous peroxidase activity was blocked with 3% H_2_O_2_ and non‐specific binding was blocked with 2% BSA. Primary antibodies for OPG (ab124820; Abcam, Cambridge, UK), RANKL (ab216484; Abcam), and SOST (AF1589; R&D Systems, Minneapolis, MN, USA) were applied for 1 h at 37°C. Immunohistochemical detection of sclerostin using the AF1589 antibody was performed as previously described (Nakashima et al. 2024). Detection was performed using ImmPRESS Polymer Reagents and DAB substrate, with hematoxylin counterstaining. Negative controls included isotype IgG or omission of primary antibody. All histological and immunohistochemical sections were digitally scanned using the Motic EasyScan digital slide scanner (Motic, Xiamen, China; software version 1.0.4.105b) at ×20 magnification, and subsequently analyzed using ImageJ software by a blinded examiner using a consistent threshold applied across all samples (see Figure legends for detailed quantification methods).

### 2.6 Statistical Analysis

Data are presented as mean ± standard deviation (SD). Group comparisons were performed using the Kruskal–Wallis test with Dunn's multiple comparisons test. Statistical significance was set at *p* < 0.05. Analyses were conducted using GraphPad Prism 8.4.3 (GraphPad Software, San Diego, CA, USA). All analyses were performed by investigators blinded to group allocation.

## 3 Results

### 3.1 Clinical Observation and Micro‐Computed Tomography (μCT)

To evaluate the clinical and radiographic features of extraction socket healing, gross examination and three‐dimensional micro‐CT analysis were performed 3 weeks after maxillary molar extraction.

On clinical examination, except for limited areas of exposed necrotic bone observed in the Dmab group, overt inflammatory signs such as erythema, swelling, or purulent discharge were not evident in the other groups. Three‐dimensional μCT reconstructions demonstrated more advanced bone remodeling in the Ctrl and Rm groups, characterized by relatively blurred boundaries and trabecular continuity with the surrounding alveolar bone. In contrast, distinct socket shapes were observed in the Rm + Dmab and Dmab groups, indicating less bone remodeling (Figure 2A).

**FIGURE 2:**
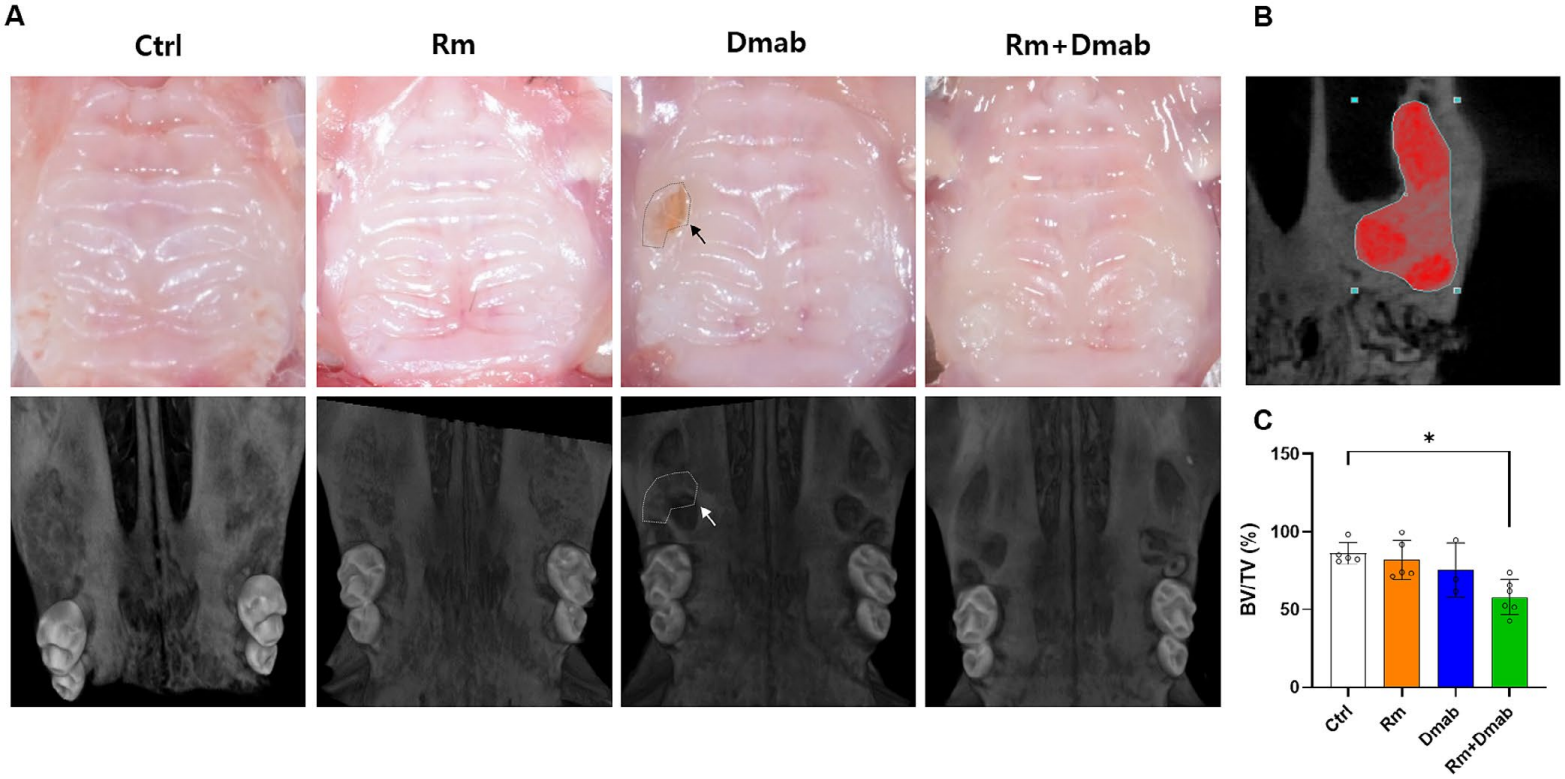
Representative intraoral photographs and corresponding three‐dimensional (3D) micro‐computed tomography (μCT) reconstructions of the maxillae harvested 3 weeks after maxillary first molar extraction in mice. (A) Intraoral clinical photographs and corresponding 3D μCT images for each of the four experimental groups. In the Dmab group, the intraoral image shows exposed necrotic bone (black arrow), and the μCT image highlights the necrotic area with a dotted outline. In contrast, the other groups showed no visibly distinct healing differences on gross examination. (B) Region of interest (ROI) on axial μCT sections encompassing the extraction socket and interradicular septum of the maxillary first molar. (C) Quantitative analysis of the bone volume to total volume (BV/TV) ratio. Kruskal–Wallis analysis revealed significant differences among groups, with the Rm + Dmab group exhibiting the lowest BV/TV. Ctrl (saline for 3 weeks); Rm + Dmab (romosozumab for 3 weeks followed by anti‐RANKL antibody for 7 weeks); Dmab (saline for 3 weeks followed by anti‐RANKL antibody for 7 weeks); Rm (romosozumab for 3 weeks). *p < 0.01.

The bone volume fraction (BV/TV ratio, %) in the extraction socket was 86.00 ± 7.00 in the Ctrl group, 81.97 ± 12.78 in the Rm group, 75.29 ± 17.23 in the Dmab group, and 58.02 ± 11.24 in the Rm + Dmab group. The Rm + Dmab group showed significantly lower BV/TV compared to the Ctrl group (𝑝 < 0.05), indicating impaired bone regeneration (Figure 2C). Although the Rm and Dmab groups also exhibited lower BV/TV than the Ctrl, the differences were not statistically significant.

These findings indicate that sequential romosozumab and denosumab treatment significantly impaired bone regeneration within the extraction socket, with both clinical and radiographic features consistent with MRONJ‐like changes.

### 3.2 H&E Staining

Given that micro‐CT analysis revealed impaired bone regeneration in the Rm + Dmab group, histological examination was performed to characterize the extent of bone necrosis at the cellular level.

H&E‐stained sections showed a progressive increase in empty osteocyte lacunae and disrupted bone architecture in the Rm + Dmab group compared to the Ctrl and Rm groups. The Rm + Dmab group exhibited a significantly higher number of empty lacunae (65.61 ± 23.13/mm^2^) and a significantly larger necrotic bone area (1.57% ± 0.84%) compared to the Ctrl group (26.49 ± 6.15/mm^2^ and 0.17% ± 0.21%, respectively; *p* < 0.01 for both). The Rm + Dmab group also showed a significantly higher number of empty lacunae compared to the Rm group (*p* < 0.05) (Figure 3).

**FIGURE 3:**
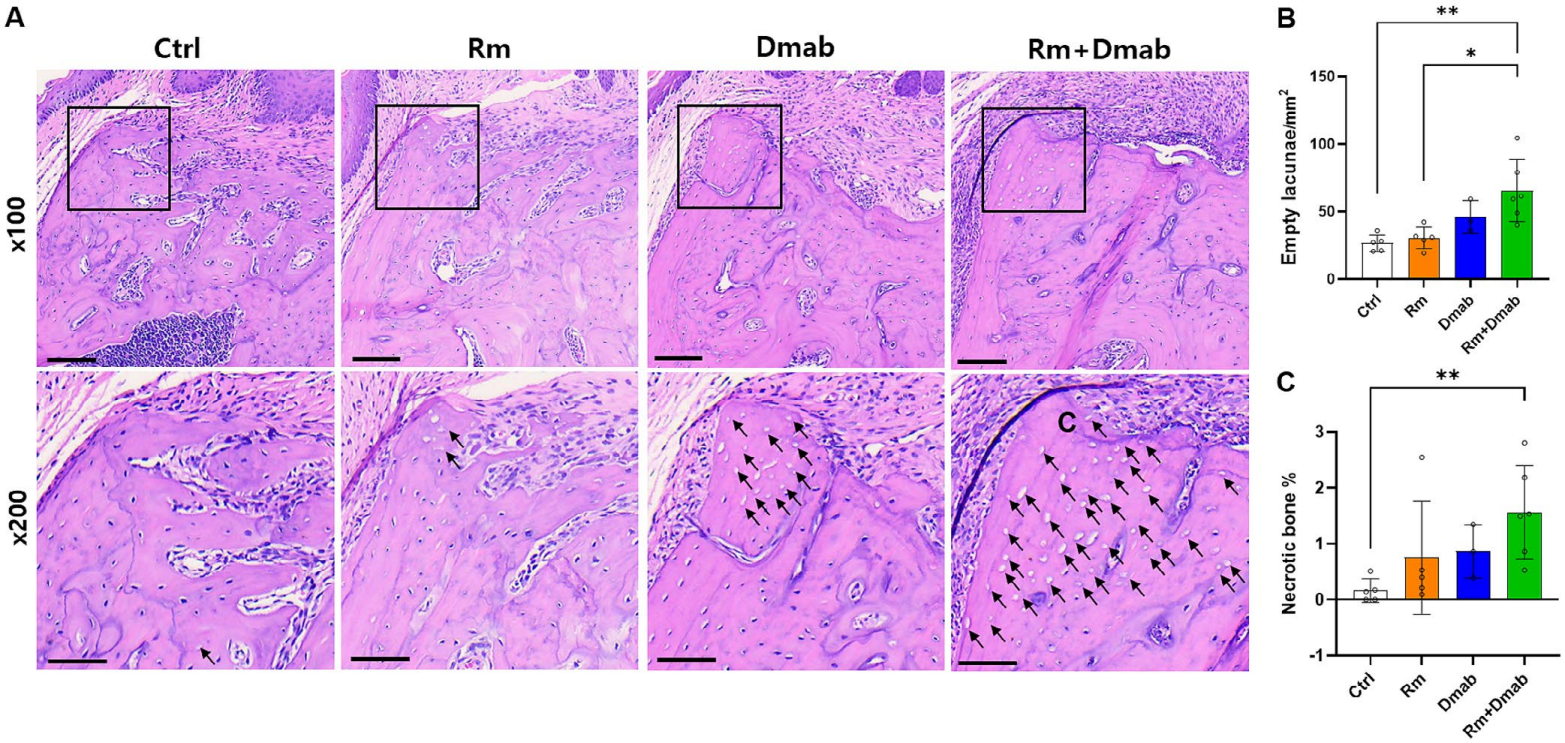
Histological analysis of bone necrosis and osteoclast activity in extraction sockets 3 weeks after maxillary first molar extraction. The top row shows low‐magnification hematoxylin and eosin (H&E)‐stained sections (×10). Boxed regions indicate areas of interest (ROIs), which are shown at higher magnification (×20) in the corresponding lower panels. (A) A progressive increase in empty osteocyte lacunae was observed from the control group to the Rm, Dmab, and Rm + Dmab groups, indicating increasing degrees of osteocyte loss and bone necrosis. Most lacunae were occupied in the control group, while clustered empty lacunae were most prominent in the Rm + Dmab group. (B) Quantification of empty lacunae per unit area (number/mm^2^). The Rm + Dmab group showed a significantly higher number of empty lacunae compared to the control and Rm groups, based on Dunn's post hoc test. (C) Necrotic bone area (%). The Rm + Dmab group exhibited a significantly larger necrotic area compared to the control group. Ctrl (saline for 3 weeks); Rm + Dmab (romosozumab for 3 weeks followed by anti‐RANKL antibody for 7 weeks); Dmab (saline for 3 weeks followed by anti‐RANKL antibody for 7 weeks); Rm (romosozumab for 3 weeks). *p < 0.05; **p < 0.01. Scale bars: 100 μm (low magnification, ×10); 60 μm (high magnification, ×20).

Collectively, these histological findings confirm that sequential romosozumab and denosumab treatment induced the greatest degree of bone necrosis, supporting the development of MRONJ‐like lesions at the extraction site.

### 3.3 TRAP Positive Cell Staining

To examine whether the observed bone necrosis was accompanied by suppression of osteoclast activity, TRAP staining was performed to quantify osteoclast numbers within the extraction socket.

TRAP‐positive multinucleated osteoclasts were markedly reduced in the drug‐treated groups compared to the Ctrl group. The Rm + Dmab group showed the most pronounced reduction, with a significantly lower number of TRAP‐positive cells (2.64 ± 0.77/mm^2^) compared to the Ctrl group (11.18 ± 3.78/mm^2^; *p* < 0.001). The Rm and Dmab single‐agent groups also showed reduced osteoclast counts relative to the Ctrl, though these did not reach statistical significance (Figure 4).

**FIGURE 4:**
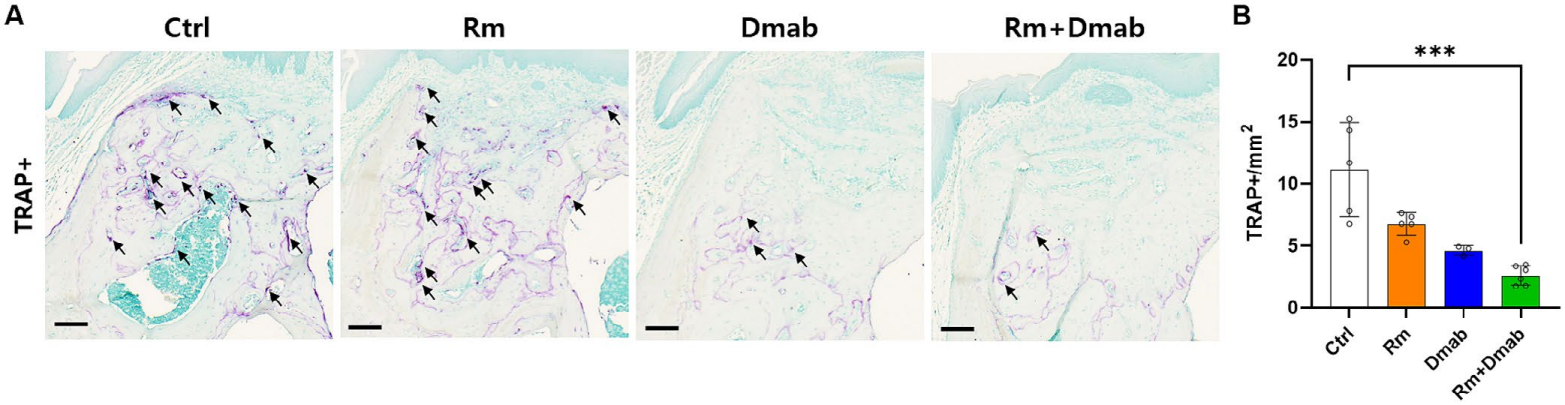
Tartrate‐resistant acid phosphatase (TRAP) staining to assess osteoclast activity in the extraction sockets 3 weeks after maxillary first molar extraction. (A) Representative TRAP‐stained sections from the control (A), Rm (B), Dmab (C), and Rm + Dmab (D) groups. TRAP‐positive multinucleated osteoclasts (black arrows) are observed along bone surfaces. (B) Quantification of TRAP‐positive cells per unit area (number/mm^2^). The number of TRAP‐positive cells was significantly lower in the Rm + Dmab group compared to the control. Ctrl (saline for 3 weeks); Rm + Dmab (romosozumab for 3 weeks followed by anti‐RANKL antibody for 7 weeks); Dmab (saline for 3 weeks followed by anti‐RANKL antibody for 7 weeks); Rm (romosozumab for 3 weeks). ***p < 0.001. Scale bars: 100 μm (low magnification, ×10).

These results demonstrate that both antiresorptive and sequential treatment regimens suppressed osteoclast activity, with the most severe suppression observed in the Rm + Dmab group, consistent with the proposed pathogenesis of MRONJ.

### 3.4 RANKL/OPG Ratio

To further investigate the molecular basis of osteoclast suppression, immunohistochemical analysis of RANKL and OPG expression was performed to assess the local bone remodeling environment.

The RANKL/OPG ratio was significantly lower in the Dmab group (0.32 ± 0.10) compared to the Ctrl group (7.26 ± 8.03; *p* < 0.05), indicating a shift toward an antiresorptive local environment. Although not statistically significant, the Rm + Dmab group similarly showed a markedly reduced RANKL/OPG ratio (0.73 ± 0.50) compared to the Ctrl group (Figure 5B).

**FIGURE 5:**
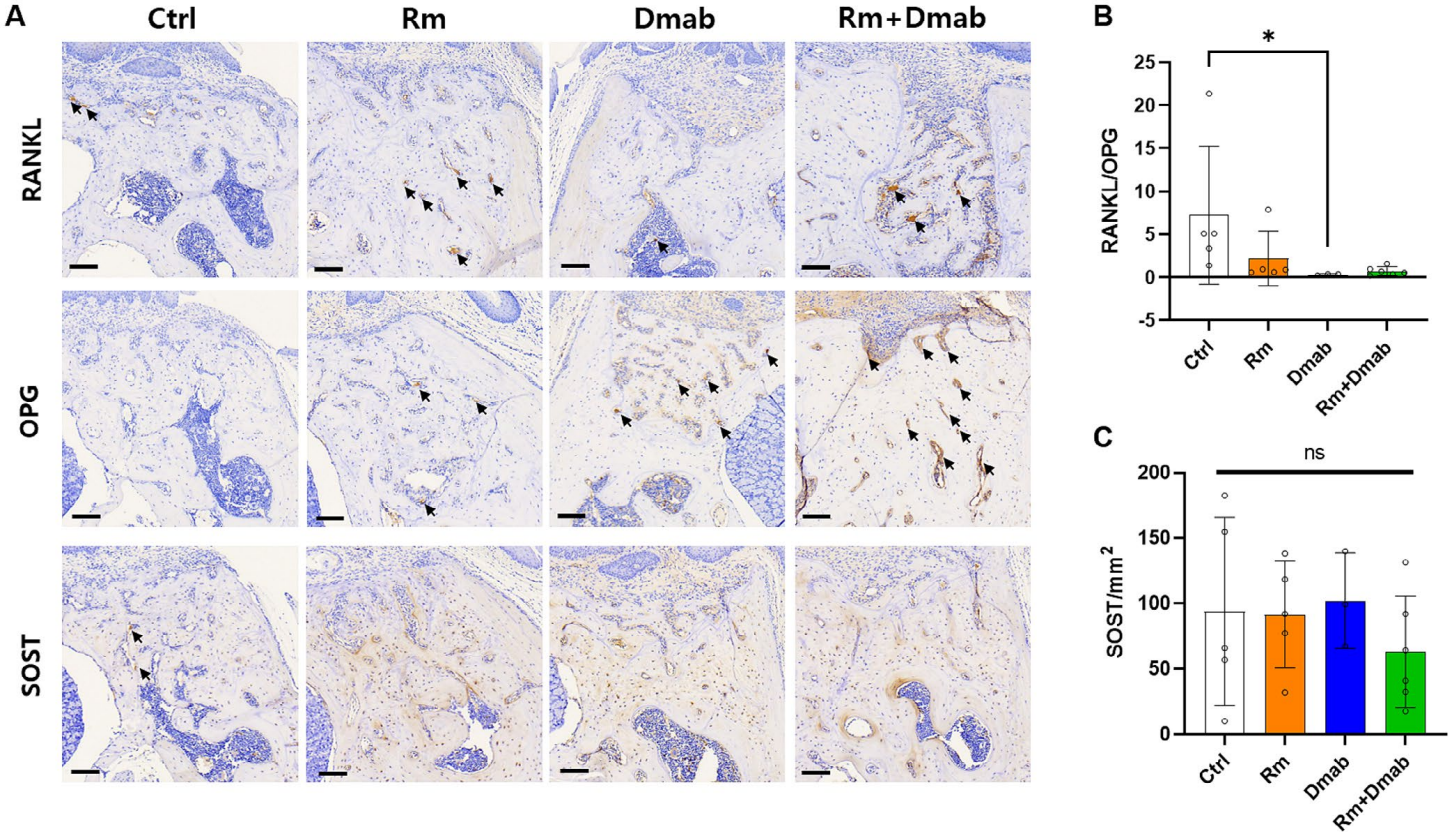
Immunohistochemical analysis of RANKL, OPG, and Sclerostin (SOST) expression in extraction sockets 3 weeks after maxillary first molar extraction. (A) Representative immunohistochemical images of RANKL‐, OPG‐, and SOST‐positive cells in the control, Rm, Dmab, and Rm + Dmab groups. Positive staining is indicated by black arrows. RANKL‐positive cells were primarily localized along the bone surface, while OPG‐positive cells appeared more diffusely distributed. SOST expression was mainly observed in osteocytes within the bone matrix. (B) Quantitative analysis of the RANKL/OPG ratio. The Dmab group showed a significantly lower ratio compared to the Ctrl group. No significant differences were observed in the RANKL/OPG ratios of the Rm and Rm + Dmab groups compared to the Ctrl. (C) Quantification of SOST‐positive cells per unit area (number/mm^2^). No significant differences were observed among groups. Ctrl (saline for 3 weeks); Rm + Dmab (romosozumab for 3 weeks followed by anti‐RANKL antibody for 7 weeks); Dmab (saline for 3 weeks followed by anti‐RANKL antibody for 7 weeks); Rm (romosozumab for 3 weeks). *p < 0.05; ns: not significant. Scale bars: 100 μm (low magnification, ×10).

These findings suggest that RANKL inhibition, particularly in the Dmab group, substantially altered the local bone remodeling balance toward reduced osteoclastogenesis, which may contribute to impaired bone healing and MRONJ pathogenesis.

### 3.5 SOST Staining

Finally, sclerostin (SOST) expression was evaluated to determine whether romosozumab treatment resulted in detectable changes in osteocyte‐derived Wnt signaling inhibition within the extraction socket.

No significant differences in SOST expression were observed among the four groups (Figure 5C). The absence of a significant reduction in SOST expression may reflect the finite duration of romosozumab administration, as sclerostin levels are known to rebound following treatment cessation. Further studies incorporating molecular quantification methods such as RT‐PCR or Western blotting are warranted to more precisely characterize sclerostin dynamics following romosozumab treatment.

## 4 Discussion

Denosumab, a human monoclonal antibody widely used for osteoporosis and certain bone tumors, effectively inhibits bone resorption by binding to RANKL (Kostenuik et al. 2009; McClung et al. 2006). However, its association with MRONJ has been documented since 2010 (Taylor et al. 2010). Animal models of MRONJ have often combined tooth extractions with high‐dose bisphosphonates to mimic human disease (Allen and Ruggiero 2014; Kikuiri et al. 2010). Since denosumab does not bind to mouse RANKL, conducting animal studies to understand its effects in standard mice is challenging. To overcome this, researchers developed humanized RANKL knock‐in mice (Kostenuik et al. 2009) or used mouse anti‐RANKL monoclonal antibodies (mAb) to mimic the effects of denosumab in mice. Such approaches increase bone mass and bone mineral density (BMD) in mice, comparable to the effect of denosumab in humans (Furuya et al. 2011; Kuroshima et al. 2016). Furthermore, Kuroshima et al. demonstrated that murine anti‐RANKL mAb treatment delays oral osseous wound healing and sustains inflammation in soft tissue, findings consistent with the MRONJ‐like lesions observed in the present study. In our study, we utilized a murine anti‐RANKL monoclonal antibody as a denosumab‐equivalent surrogate to investigate how RANKL inhibition affects post‐extraction alveolar bone healing.

Romosozumab, a newer anabolic agent, targets and inhibits sclerostin, an osteocyte‐derived protein that negatively regulates bone formation (Hsu et al. 2022). Unlike bisphosphonates and denosumab, romosozumab activates Wnt/β‐catenin signaling to stimulate osteoblastic activity and bone formation, while also modestly reducing bone resorption (Lim and Bolster 2017). Some reports have suggested that romosozumab use may increase the risk of osteonecrosis of the jaw (ONJ) (Palla et al. 2023; Peng, Wang, Liu, Zhang, and Zhang 2022; Ruggiero et al. 2022). The 2022 American Association of Oral and Maxillofacial Surgeons (AAOMS) Position Paper classified romosozumab, along with bisphosphonates and denosumab, as a therapy potentially associated with elevated ONJ risk (Ruggiero et al. 2022). Additionally, the US Food and Drug Administration (FDA) adverse event reporting system linked romosozumab to ONJ, although with a much lower incidence than bisphosphonates (Peng, Wang, Liu, et al. 2022). In clinical trials, romosozumab significantly reduced fracture risk; among 5376 patients treated with romosozumab in two phase III trials, only three cases of MRONJ were reported (Cosman et al. 2016; Saag et al. 2017). The mechanism by which romosozumab might contribute to ONJ is unclear, but there are case reports of severe ONJ in patients on romosozumab (Palla et al. 2023). ONJ pathogenesis in patients on antiresorptives is more likely due to suppressed bone resorption rather than impaired bone formation (Lee et al. 2025). Indeed, the 2024 Italian MRONJ position paper concluded that the contribution of romosozumab to ONJ risk remains uncertain, given that most ONJ cases occurred in patients with prior exposure to bisphosphonates or denosumab (Bedogni et al. 2024). A previous experimental study in a periodontitis mouse model found no evidence of ONJ development following romosozumab administration (Nakashima et al. 2024), and another study showed that sclerostin deficiency did not cause ONJ but instead reduced the risk of MRONJ by enhancing bone formation and wound healing in the extraction socket (Hadaya et al. 2019).

In our study, we employed a sequential treatment (romosozumab followed by denosumab) in line with AACE guidelines for managing patients at very high fracture risk (Camacho et al. 2020). This design allowed us to evaluate the effects of romosozumab and denosumab individually and in combination, reflecting a real‐world therapeutic strategy. We aimed to clarify each agent's contribution, as well as any synergistic effects, on MRONJ development after tooth extraction.

Bone exposure with signs of necrosis was observed only in the Dmab group, and other inflammatory features such as swelling and pus discharge were not prominent. Micro‐CT analysis of the extraction sites revealed that the BV/TV was significantly lower in the Rm + Dmab group than in the Ctrl group (*p* < 0.05, Figure 2). Although the Rm + Dmab group had lower BV/TV values than the Dmab group, the difference was not statistically significant. Our findings parallel previous findings by Aghaloo et al. (2014) and de Molon et al. (2015) who reported that RANKL inhibition through agents such as anti‐RANKL antibodies or OPG‐Fc can preserve or increase bone volume while simultaneously impairing normal bone remodeling and healing, a combination that predisposes to ONJ. This mechanism may explain why the Dmab group in our study did not exhibit a significant BV/TV reduction compared to the control, as the early preservation of bone volume by RANKL inhibition does not necessarily reflect normal bone regeneration or healing.

Histologically, the Rm + Dmab group showed the greatest number of empty osteocyte lacunae and the largest necrotic bone area, followed by the Dmab group (Figure 3). The necrotic bone area in the Rm + Dmab group was significantly greater than in the Ctrl group (𝑝 < 0.01), while the Dmab and Rm groups did not differ significantly from the Ctrl group on post hoc analysis.

Consistent with the antiresorptive mechanism, osteoclast numbers were markedly reduced by treatment. TRAP staining revealed a significantly lower number of TRAP‐positive cells in the Rm + Dmab group compared to the Ctrl (𝑝 < 0.001) (Figure 4). The Rm and Dmab single‐agent groups also showed reduced osteoclast counts relative to controls, though these did not reach significance. Diminished osteoclast activity aligns with the proposed pathogenesis of MRONJ, wherein excessive suppression of bone resorption impairs bone turnover and healing (Buranaphatthana et al. 2021; Gross et al. 2017).

Immunohistochemical findings further supported a suppressed bone turnover state. The RANKL/OPG ratio in the Dmab group was significantly lower than the Ctrl group (𝑝 < 0.05) (Figure 5), indicating a shift toward an antiresorptive local environment. The Rm + Dmab group similarly showed a low RANKL/OPG ratio, though this did not reach statistical significance compared to the Ctrl group. The RANKL/OPG signaling axis plays a crucial role in osteoclastogenesis and bone remodeling, and alterations in this ratio have been linked to impaired bone remodeling and MRONJ development in both patients and animal models (de Molon et al. 2015; Di Nisio et al. 2015; Lewiecki and Bilezikian 2012). Our findings are in line with this concept and indicate that RANKL inhibition shifts the RANKL/OPG balance toward reduced osteoclastogenesis and bone resorption.

We observed no significant differences in sclerostin (SOST) expression among the groups (Figure 5). IHC‐based assessment of SOST expression using the AF1589 antibody (R&D Systems) is an established approach in MRONJ‐related research and has been employed in previous studies investigating sclerostin in the context of jaw osteonecrosis (Nakashima et al. 2024). This result contrasts with findings by Nakashima et al. who reported that the genetic absence of sclerostin reduced ONJ risk in mice. A possible reason for the discrepancy is the duration of SOST suppression: their SOST‐knockout model represents lifelong sclerostin deficiency, whereas our mice received a finite course of romosozumab, and by the 3‐week post‐extraction mark, sclerostin levels may have partially rebounded. Indeed, prior studies indicate that the pharmacological effect of sclerostin antibodies diminishes over time, with SOST expression returning to baseline several weeks after treatment cessation (Holdsworth et al. 2018; Morrell et al. 2021). Thus, differences in timing and persistence of sclerostin inhibition could explain the divergent outcomes. Nevertheless, molecular quantification methods such as RT‐PCR or Western blotting would allow more precise characterization of sclerostin dynamics following romosozumab treatment, and this represents an important direction for future investigation.

Although both antiresorptive agents targeting RANKL and anabolic agents such as romosozumab achieve their primary goals of increasing bone density and reducing fracture risk, our findings suggest that using them sequentially may compromise bone healing after invasive dental procedures. Clinically, this highlights the need for caution in managing high‐risk osteoporosis patients who might undergo tooth extraction or similar oral surgical procedures. Our study has several limitations. First, as an exploratory pilot study, the sample size was limited, which warrants cautious interpretation of the findings. Despite this limitation, the findings were consistent across multiple independent outcome measures, including micro‐CT, histomorphometric, and immunohistochemical analyses, all pointing in the same direction, thereby supporting the biological plausibility of the observed effects. Second, the drug administration periods intentionally differed across groups to reflect the clinical sequential therapy protocol recommended by the 2020 AACE guidelines for patients at very high risk of osteoporotic fracture (Camacho et al. 2020), in which an anabolic agent is administered prior to transitioning to antiresorptive therapy. While this design enhances clinical translational relevance, it precludes direct comparisons of drug duration effects between groups, and future studies incorporating duration‐matched control groups would be valuable to isolate the contribution of sequential exposure from total treatment duration. Third, the anabolic effect of romosozumab may have been less pronounced in mice than expected, which could have influenced the observed outcomes. Finally, rodent bone turnover rates differ substantially from those of humans, and these findings may not directly translate to the clinical setting. Therefore, further research with larger sample sizes and possibly larger animal models or clinical data are necessary to validate these findings and to establish strategies that maximize therapeutic efficacy while minimizing the risk of MRONJ.

In conclusion, sequential romosozumab followed by denosumab therapy was associated with impaired post‐extraction bone healing and an increased risk of MRONJ. These findings highlight the need to elucidate the mechanistic basis of this adverse effect and to develop strategies to mitigate the associated risk. Further research is essential to ensure that patients can receive the bone‐strengthening benefits of these therapies without compromising oral surgical outcomes.

## Author Contributions

**Hae‐Seo Park:** investigation, formal analysis, visualization, writing – original draft. **Moon‐Young Kim:** conceptualization, funding acquisition, methodology, supervision, writing – review and editing. **Seo‐Yeong An:** investigation, formal analysis, visualization, writing – original draft. **Reuben H. Kim:** writing – review and editing.

## Funding

The present research was supported by the research fund of Dankook University in 2025.

## Ethics Statement

The study was approved by the Dankook University Institutional Animal Care and Use Committee (DKU‐23‐004), and all procedures adhered to the relevant regulations for animal experimentation.

## Consent

The authors have nothing to report.

## Conflicts of Interest

The authors declare no conflicts of interest.

## Data Availability

The data that support the findings of this study are available from the corresponding author upon reasonable request.
